# Impaired Rod-Mediated Vision is the Functional Hallmark of Ageing and Early and Intermediate Age-Related Macular Degeneration

**DOI:** 10.1007/s44402-026-00124-1

**Published:** 2026-06-22

**Authors:** Cynthia Owsley, Christine A. Curcio

**Affiliations:** Department of Ophthalmology and Visual Sciences, Heersink School of Medicine, University of Alabama at Birmingham, Birmingham, Alabama USA

**Keywords:** Aging, Age-related macular degeneration, Scotopic sensitivity, Rod-mediated dark adaptation, Rods, Cones

## Abstract

Laboratory and patient-oriented research on photoreceptors and vision in ageing and early and intermediate age-related macular degeneration (AMD) were conducted in parallel starting in the 1990s by Christine Curcio and Cynthia Owsley, respectively. They joined forces in two longitudinal observation studies, the Alabama Study on Age-related Macular Degeneration (ALSTAR) in 2009 and in ALSTAR2 in 2019, together involving >1100 participants. These studies established rod-mediated dark adaptation (RMDA) measured close to the fovea as the first functional biomarker for incident early AMD and the progression of AMD. These studies used standardised grading of colour fundus photography and prioritised large samples for statistical power. RMDA is a global measure of dysregulated transport between the circulation and photoreceptors, involving at least seven different steps in a retinoid re-supply route especially needed by rods, and a surrogate for the delivery of other essentials across this route. The choice of functional and imaging outcome measures was informed by a model of AMD pathophysiology based on drusen biology, as discovered in the laboratory using high-quality human donor eyes. Specifically, high-risk drusen in the central retina were thought to result from large lipoproteins constitutively made by the retinal pigment epithelium and impaired in transit to circulation by ageing changes in Bruch’s membrane and choriocapillaris. Imaging studies in ALSTAR/2 thus included optical coherence tomography (OCT) angiography assessments of choriocapillaris flow signal and OCT assessments of outer bands involved in intercellular transfer (interdigitation zone). Of seven vision tests utilised in ALSTAR2, only RMDA achieved a Minimum Clinically Important Difference at 3 years follow-up. This research highlights the importance of developing functionally valid structural endpoints for use in early and intermediate AMD intervention trials. Research to date supports the idea that functional changes emerge earlier than structural changes in early and intermediate AMD. Clinicaltrials.gov # NCT04112667 (registration date October 7, 2019)

Key Points

- Unbiased photoreceptor counts in human donor eyes in ageing and age-related macular degeneration revealed vulnerable rods surrounding a centre of protected cones, attributed to mechanisms of dysregulated transport also contributing to high-risk drusen formation, under the fovea.
- The selective vulnerability of rod photoreceptors in ageing and early and intermediate age-related macular degeneration, established three decades ago, prompted us to study rod-mediated vision in these populations, demonstrating that rod-mediated sensitivity loss was the first functional biomarker of disease onset.
- Through ALSTAR and ALSTAR2 we demonstrated that rod-mediated dark adaptation in ageing and early age-related macular degeneration was associated with both the incidence and progression of age-related macular degeneration 3 years later; associations that had cross-sectional and longitudinal associations with structural changes in the retina.

## Introduction

Age-related macular degeneration (AMD), a leading cause of irreversible vision impairment in older adults in the United States and other countries [1–8], is a disease of the photoreceptor support system involving the retinal pigment epithelium (RPE), Bruch’s membrane, and choroid, impacting retinoid re-supply, and leading to photoreceptor demise and vision loss [9]. AMD is a complex, multifactorial disease of ageing in which central photoreceptors are lost by a neovascular event or an atrophic process. AMD generates significant personal burden, including reading, driving, and face perception difficulties, depression and anxiety [10–14]. Currently, there are neither proven means to arrest the progression of early AMD nor prevention strategies for those at high risk [15, 16]. A barrier to developing preventative strategies is a limited understanding of how ageing transitions to early AMD. Equally important is the lack of valid and responsive functional endpoints for evaluating hypothesised early interventions that are acceptable to the US Food and Drug Administration (FDA), the European Medicines Agency (EMA), and the scientific community [17, 18]. Anatomical endpoints are favoured due to the speed, objectivity, and comprehensiveness of modern imaging. However, the most promising anatomical endpoints will be those that correlate with visual function, facilitating their use as primary outcome or surrogate measures [19]. In addition, visual function endpoints are what personally matter to patients during their everyday lives.

About three decades ago, Curcio and colleagues [20] demonstrated that during ageing, rods in the central retina die before cones, suggesting that rods are selectively vulnerable to degeneration, whereas cones are resilient. This trend continues into early and intermediate AMD [21], where cone-mediated function, such as visual acuity, is excellent and relatively stable [22, 23], whereas rod-mediated function degenerates over this time scale [24–26]. The last surviving photoreceptors in late AMD are cones, not rods [21].

The selective tissue-level vulnerability of rod photoreceptors in ageing and AMD prompted us three decades ago to focus on rod-mediated vision. Specifically, we sought to determine if rod-mediated vision was also selectively vulnerable in ageing and early and intermediate AMD. In this article, we will describe the major scientific milestones in this collaborative journey. In a previous article based on our Proctor Medal Lecture [27], we discuss more fully how impairments in rod-mediated vision relate to structural biomarkers visible in clinical imaging.

## Histologic Basis for Investigating Rod Vision

Three histologic studies [20, 21, 28] on photoreceptors in ageing and AMD and two later studies on the RPE [29, 30] laid the groundwork and supported in parallel the visual function research described throughout this review. These studies are summarised here to provide a biological context for the functional research as it unfolded. In actuality, the laboratory and patient-oriented research were conducted in parallel starting in the early 1990s.

**Fig. 1:**
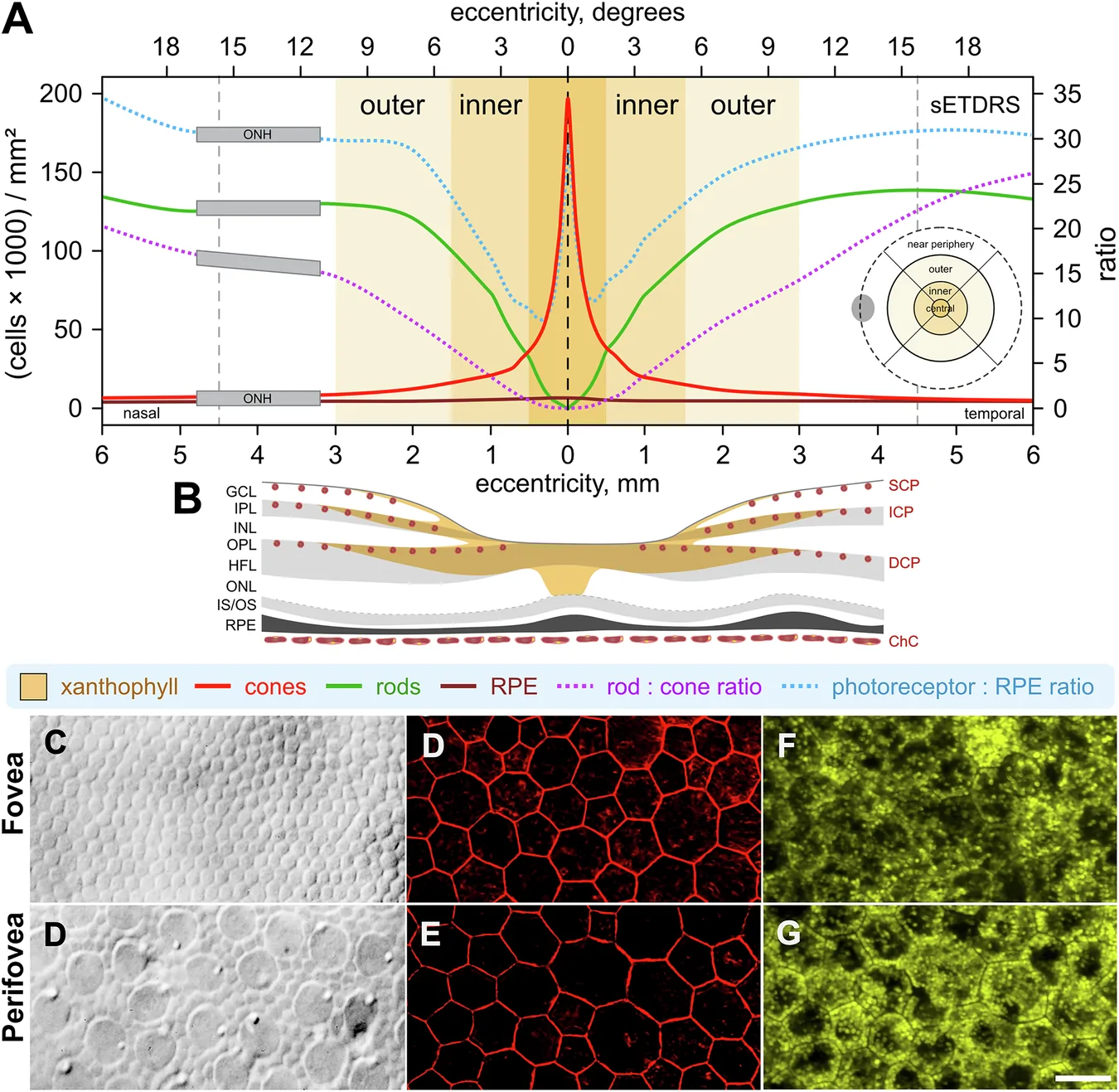
Spatial density and morphology of photoreceptors and RPE in human retina. **A** Spatial density of cones, rods, retinal pigment epithelium (RPE) cells along the horizontal meridian of young adult human retina. Rods and cone inner segments were counted in the same flat-mounted tissues; rod: cone ratios were calculated directly [28]. RPE was counted in separate eyes using similar methods [29]. Photoreceptor: RPE ratios were computed by matching eccentricities and by assuming for simplicity that each RPE cell tends to the photoreceptors directly above it [131]. The grading grid of the Early Treatment of Diabetic Retinopathy Study (ETDRS) [38] is at the right. Outer diameters of the outer ring, inner ring, and central subfield are 6, 3, and 1 mm, respectively, and are not drawn at the same spatial scale as the main graph. A ‘supplemented’ ETDRS grid (sETDRS) includes an additional ring with inner and outer diameters of 6 and 9 mm, respectively encompassing the near periphery region to capture high rod densities. Eccentricities on the graph and rings in the grid are colour-coded to match the concentration of xanthophyll carotenoid pigment, shown in projection view (**A**) and cross-sectional view (**B**). Central subfield and inner ring together comprise the macula lutea and exhibit the highest and next-highest population-level risk, respectively for age-related macular degeneration (AMD) progression. ONH, optic nerve head. **B** Schematic cross-section of a human fovea, with vascular plexuses and xanthophyll pigment indicated. Xanthophyll carotenoid pigment (yellow) is schematised [132] from microdensitometry of sections through a macaque monkey fovea [133]. Abbreviations (top to bottom) - *Cells:* GCL, ganglion cell layer; HFL, Henle fibre layer; INL, inner nuclear layer; IPL, inner plexiform layer; IS/OS, inner and outer segment of photoreceptors together; ONL, outer nuclear layer; OPL, outer plexiform layer; OS, outer segment; RPE, retinal pigment epithelium. *Vascular plexuses:* SCP, superficial capillary plexus; DCP, deep capillary plexus; ICP, intermediate capillary plexus; ChC, choriocapillaris. A conversion factor of 0.288 mm/° of visual angle is used. **C**, **D** Mosaic of photoreceptor inner segments at the ellipsoid level, captured by video-enhanced differential interference contrast microscopy. Fovea has only cones. Perifovea has threefold larger cones surround by complete rings of rods. **E**, **F** RPE cytoskeleton labeled by Alexa 647-tagged phalloidin, captured by epifluorescence microscopy. RPE cells have a precisely polygonal cytoskeleton with straight edges and sharp vertices. **G**, **H** Autofluorescence emissions after 488 nm excitation in the same cells as **E**, **F**. The fovea has a higher proportion of hexagonal cells and weaker autofluorescence emissions at this wavelength (**C**, **E**) than cells in the perifovea (**D**, **F**). Tissues are from white donors, >80 years of age. Scale bar, 10 µm, applies to all panels. Figures were reprinted under Creative Commons license [32] with modification.

Photoreceptors and RPE can be viewed microscopically, in flat mounts of excised tissues (Fig. 1C–G). Together, photoreceptors and RPE form an integrated light-sensing chip for the brain, like the chip in a smartphone. The highly compartmentalised photoreceptors connect via bipolar cells and ganglion cells to multiple pathways to the brain. The fovea contains the highest packing density of cone inner segments, in an area also lacking rods (Fig. 1A, B). Foveal vision, when utilised in activities like naturalistic reading, activates vast portions of the brain [31]. Photoreceptors and RPE are supported by a microvasculature, the choriocapillaris. This is the terminal capillary bed of the choroid, a specialised macrovasculature of the systemic circulation. The inner boundary of the choroid (Bruch’s membrane) is a planar vessel wall.

These studies provided continuous, two-dimensional digital maps of the sensing chip, in and around the fovea and an elliptical ring of high rod density near the arcades. In the highly rod-dominant human retina (rod: cone, 20:1 [32]), key observations were made about ageing in the central retina. These are: (1) preservation of foveal cone density (rod: cone 0.4:1 [32]) and modest loss (11%) outside the fovea, (2) ~36% loss of rods in an annulus around the fovea (5°; rod: cone 4:1 [32]) that was exacerbated in early stages of AMD, (3) proportionately less loss at the rod ring (12°; rod: cone 13:1), and (4) preservation of RPE cell density in ageing despite gain of autofluorescence. Together with other large series of human RPE counts, including a 1996 unpublished series from the histology laboratory, these collectively showed no strong effect of ageing on RPE cell populations in the central retina [33–35].

Several qualities enabled the mapping studies to continue informing visual function research over the years. First, sample locations for cell counts were unbiased, covering the whole retina for photoreceptors, importantly including both the fovea and surrounding areas, despite early thinking that AMD impacted primarily foveal cones [36]. Second, unstained tissues visualised with differential interference contrast optics utilised gradients of refractive index to generate contrast and visualise subcellular detail such as mitochondria in the inner segment ellipsoid (Fig. 1C, D). This experience presaged tissue-level optical considerations for optical coherence tomography (OCT) [37]. Other innovations included dissection methods designed to capture accurately the location of foveal RPE and a fovea-centred coordinate system allowing alignment and comparison across eyes and across cell layers (Fig. 1A, B), again presaging depth-resolved OCT. The continuous two-dimensional maps facilitated translation to clinical use via the Early Treatment of Diabetic Retinopathy Study (ETDRS) grid [38].

Other major efforts at quantifying human photoreceptors in ageing in the 1990s did not capture the features that ultimately proved important for AMD functional outcomes. Gao and Hollyfield [33] counted inner segments in the foveal rod-free area and rod-dominant equatorial retina. Foveal cone densities were highly variable, overlapping with our values. Rods next to the fovea were not assessed (selection bias). Panda-Jonas et al. [39–41] reported photoreceptor density in 5 mm diameter tissue punches. This measure averages across the steep density gradients of the foveal singularity and thus misses the AMD-vulnerable parafovea (spatial imprecision).

**Fig. 2:**
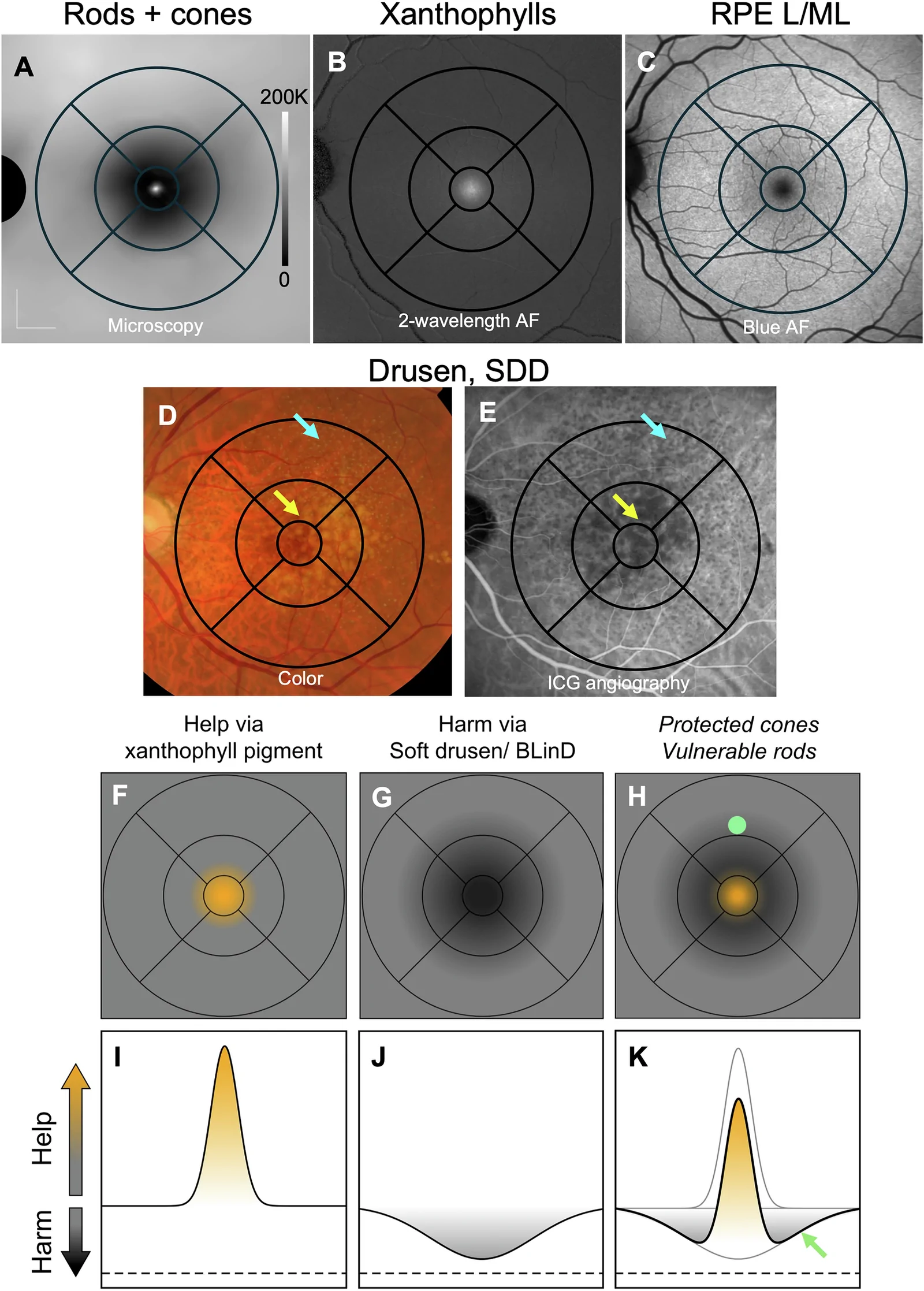
Centre-surround model aligns topographies of outer retinal cells, age-related macular degeneration (AMD) deposits, and rod-mediated dark adaptation (RMDA). **A**–**E** Fundus view, with overlay of the classic Early Treatment of Diabetic Retinopathy Study (ETDRS) grid. **A** Total photoreceptors (rods + cones)/ mm^2^ in young adult retina. Cone density peaks in the foveal centre. Rod density is highest in a broad elliptical ring near the vascular arcades. Grey-scale contour map was generated from cell counts in flat-mounted tissue. **B** Macular xanthophyll carotenoids exhibit high concentration at the foveal centre and a less steep decline with eccentricity than cones in **A** due to the presence of these compounds in inner retinal layers. Two-wavelength autofluorescence imaging is log_10_ ratio of green emissions to blue emissions, normalised by emissions at 9° eccentricity, where xanthophylls are negligible. **C** Blue autofluorescence (excitation at 488 nm) derives from retinal pigment epithelium (RPE) lipofuscin and melanolipofuscin (L/ML), secondary to daily ingestion of outer segments. Signal is low but non-zero in the foveal centre and rises to a ring of high signal in parallel with the numerically dominant rods. **D**, **E** High-risk (soft) drusen (yellow arrows) and subretinal drusenoid deposits (SDD, turquoise arrows) appear preferentially in areas with differing photoreceptor content. Colour reflectance image shows many large yellowish drusen with indistinct borders in the inner ring and smaller punctate and regularly spaced SDD at the outer ring and outside the grid. **E** Drusen and SDD are hypocyanescent on indocyanine green angiography. This dye is visualised because it is carried on plasma lipoproteins that are taken up at receptors on the basal RPE. Hypocyanescence occurs where this process is interrupted. **F**–**K** Centre-Surround model of cone resilience and rod vulnerability in ageing and early stages of AMD based on the difference of 2-dimensional Gaussian surfaces [134, 135]. **F**–**H** *en face* view of macular xanthophyll pigment (help, orange) and the Oil Spill (visible drusen and invisible basal linear deposit; harm, grey). **I**–**K** Help and harm are plotted as positive and negative directions, respectively, on one vertical axis. **I** Xanthophyll carotenoids in foveal cones and specialised Müller glia form a narrow centre. **J** The Oil Spill forms a broader circular area that is concentric with the fovea. **K** Together, help and harm make a narrow centre of foveal cone resilience atop a broad surround of para- and perifoveal vulnerability. In the surround, rods are most affected due to their exclusive dependence on the choroid, whereas cones are also supported via Müller glia from the retinal circulation. Rod mediated dark adaptation at 5° probes vision at the rim of this high-risk area (green circle and arrow). Original data: histology [28], 2-wavelength and blue autofluorescence [136], colour and indocyanine green angiography courtesy of N. Ueda-Arakawa MD (original data [137]; centre-surround model [32]).  AF autofluorescence, BLinD pre-basal linear deposi.

Other concepts from the 1980s to 1990s did profoundly influence the direction of laboratory work, ultimately contributing to a distinctive pathophysiology model called Centre-Surround that underlies our two longitudinal studies (Fig. 2). Major impetus came from a seminal histochemical study by Pauleikhoff et al. showing marked accumulation of oil red O binding lipid with age in normal Bruch’s membrane [42] (esterified cholesterol [43], triglyceride, free fatty acids, vitamin A esters). These authors wished to understand why fluid-filled pigment epithelial detachments resolved slowly in older persons and postulated a barrier to outward flow of fluid pumped by the RPE. The vascular barrier model was attractive, as it linked to prior findings of histochemically similar lipid in drusen [44]. It further suggested a potential molecular commonality of AMD with atherosclerotic cardiovascular disease, which also features age-related lipid deposition in vessel walls, despite differences between Bruch’s membrane and arterial intima in vascular morphology, hemodynamics, and participating cells. In work reviewed elsewhere [45–50], laboratory studies employing histopathology, histochemistry, electron microscopy, lipid profiling, and gene expression of human eyes strongly supported the idea that the principal oil red O binding lipid was esterified cholesterol within lipoprotein particles bearing apolipoproteins B, E. Lipoproteins are thought to be made by the RPE throughout life to release unneeded lipids to the circulation as part of normal physiology. These are impeded in transit across ageing Bruch’s membrane and choriocapillaris, eventually building up to form an ‘Oil Spill’ of visible drusen and invisible basal linear deposit in older persons. The lipoprotein pathway has been confirmed in mice, with a cell-type-specific deletion, confirming its importance in mammalian outer retinal physiology [51].

Foveal vision is important for daily activities meaningful to AMD patients, such as reading and face recognition. The fovea can be conceived as key evolutionary biology of Haplorrhine primate retina, including that of humans, which features excellent stereoscopic vision and depth perception, in addition to high visual acuity and trichromatic colour vision [52]. Foveal cones maintain very long outer segments, have high energy demands, and are accompanied by specialised Müller glia [53–56]. Accordingly, the consequences of lifelong sustenance of the fovea represent an organising principle of AMD pathophysiology. Indeed, reexamination of population-based epidemiology data for spatial determinants of progression risk pointed to drusen under the fovea as an exceedingly potent predictor of future advanced disease [57–61]. Thus, early parafoveal rod loss in ageing and early AMD stages may serve as an early warning system for the endangerment of foveal cones caused by impaired transport from the circulation. Foveal cones themselves are largely spared until late in the disease, plausibly because of protection by Müller glia, a rich reservoir of antioxidant xanthophylls and other essential support services.

## Scotopic Sensitivity: Ageing

Given the vulnerability of rod photoreceptors during ageing, we, along with our graduate student and later faculty colleague, Gregory Jackson, became interested in impairments in scotopic light sensitivity in older adults in normal macular health, as compared to young adults. For the first time in the psychophysics literature, we implemented a fundus photograph grading system to document macular health in older eyes. This approach was recommended by Samuel Jacobson, MD, PhD, a pioneer in functional and imaging outcome measure development for inherited retinopathies and a collaborator in our early AMD function studies. Use of a fundus photograph grading system was a critical step forward for designing studies of ageing vision. The grading system provides an objective definition of macular health. In contrast, the clinical impression of AMD presence and severity varies widely among clinicians. An objective and repeatable method allows comparison among published studies that also used this classification system. Fundus classification systems had already been developed and used in epidemiological studies on ageing and AMD [62, 63], but had not been previously used in psychophysical studies. In our study [64], we used a system based on the International Classification System [63] for categorising AMD into severity levels and whether the eye was in normal macular health. Our findings were that older eyes in relatively good macular health exhibited a 0.5 log unit loss in scotopic sensitivity near the fovea at 4° and 7°, both nasally and temporally [64], which was consistent with Curcio’s finding of greater rod density loss near the fovea of older eyes, corresponding to 1.8° to 10.6° [20].

We were interested in the developmental course of this scotopic sensitivity deficit during adulthood, whether it only appears late in adulthood or emerges gradually. We measured both scotopic and photopic (cone-mediated) sensitivity in 94 adults ranging in age from their 20s to the 80s (years), with light sensitivity measured at 27 test loci in the 18° radius field of the macula (fovea was not tested) [65]. Again, we used the same fundus classification system as above to identify older adults in normal macular health [63]. In plots of light sensitivity as a function of age, the best model was a linear fit. Averaging light sensitivity across all test targets, scotopic sensitivity decreased at a rate of 0.08 log units per decade, whereas photopic sensitivity decreased at half that rate per decade, 0.04 log units [65].

## Scotopic Sensitivity: AMD

**Fig. 3:**
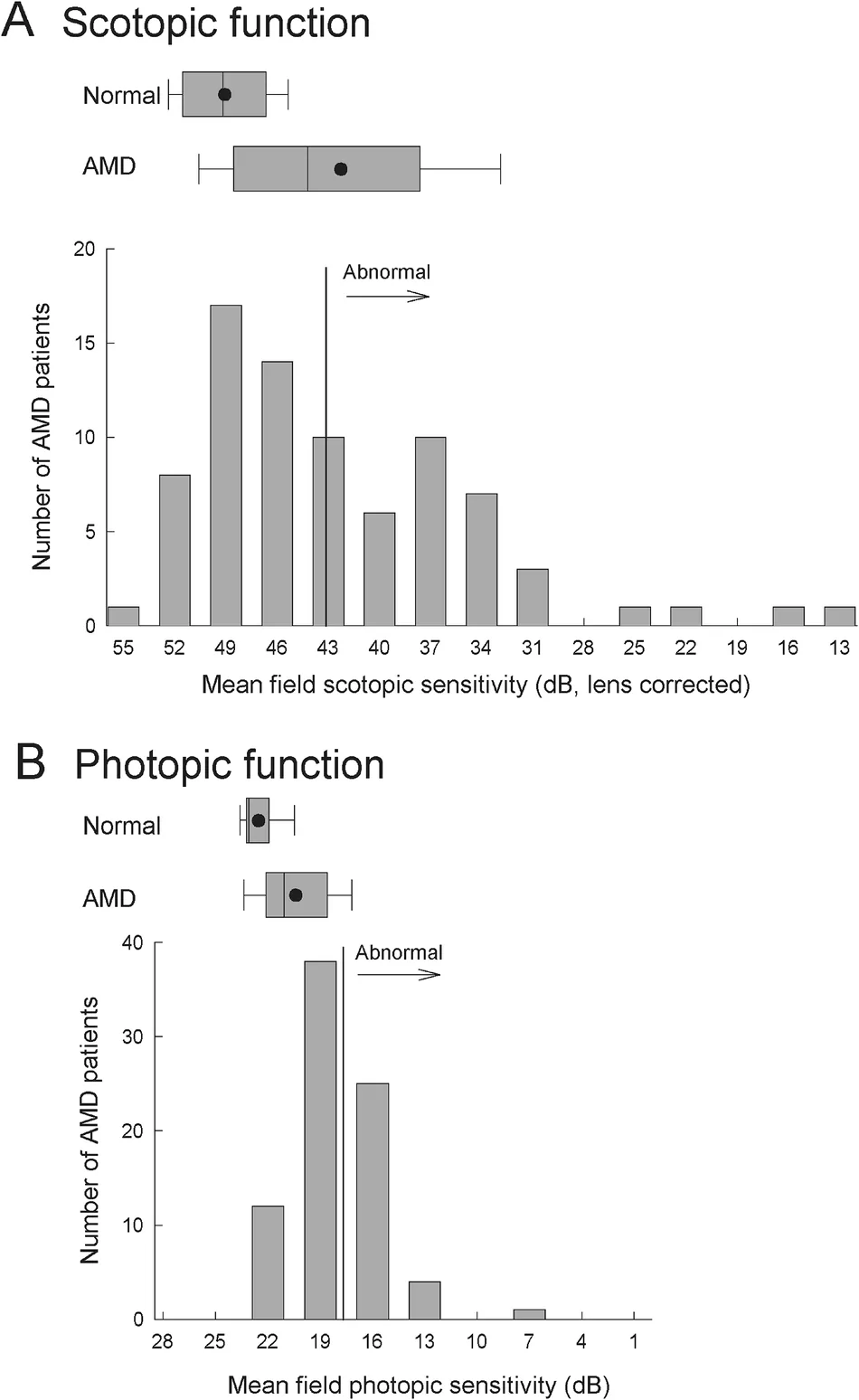
Scotopic and photopic sensitivity impairment in ageing and early age-related macular degeneration (AMD). **A** Frequency histogram of mean scotopic (dark-adapted) sensitivity for older adult eyes in normal macular health and those with age-related macular degeneration (AMD). Sensitivity is expressed in decibel (dB) units. Scotopic sensitivity is corrected for pre-retinal absorption. Box plots above each histogram compare the distributions of AMD patients to age-matched controls. Box represents 25th and 75th percentile; whiskers, 10th and 90th percentile. Solid line within the box, the median and solid circle, the mean. Vertical line in the histogram represented the lower normal limit (mean – 2 SD). **B** Frequency histogram of mean photopic (light-adapted) for older adult eyes in normal macular health and those with AMD. Units and plot representations are as described in **A** for scotopic sensitivity. Used with permission from the Association for Research in Vision and Ophthalmology through the Creative Commons with this citation [66].

A question of interest was whether we could also provide psychophysical evidence for rod vulnerability in early AMD as compared to that found in normal older adults. Collaborating with Samuel Jacobson and Artur Cideciyan [66], we measured scotopic sensitivity in 80 patients with AMD, most with early or intermediate AMD. Photopic sensitivity was also measured. Light sensitivity was measured over a 45°-diameter retinal area centred on the fovea, where 51 test locations were evaluated. Sensitivity was averaged over the 51 test targets. AMD presence and severity were based on the International Classification System. We compared their data to that of older adults in normal macular health. Results were that both scotopic and photopic sensitivity were, on average, impaired in adults with AMD compared to normals, but the sensitivity losses were more prevalent and much more severe for scotopic than for photopic sensitivity (Fig 3A, B) [66]. We also noticed that the largest scotopic sensitivity impairments were close to the fovea.

## Rod-Mediated Dark Adaptation: Ageing

Scotopic sensitivity, as discussed above, is measured under steady-state conditions in the dark, that is, testing does not begin until after the eye has adapted to darkness for approximately 45 min, so that only rod photoreceptors, not cones, are responsive to light. Another aspect of scotopic vision is dark adaptation. This is the dynamic process by which the eye recovers light sensitivity over time after exposure to a bright light (photobleach) when tested in the dark. Our focus has been on rod-mediated dark adaptation (RMDA) because rods, not cones, are vulnerable during ageing and early and intermediate AMD [20, 21]. Dark adaptation is supported by the visual cycle, which is responsible for rhodopsin regeneration [67, 68]. The first question we addressed is whether dark adaptation is perturbed in adults during the ageing process. Dark adaptometry techniques can estimate the time constants associated with the visual cycle by measuring the recovery of light sensitivity over time after exposing the photopigment to an intense light that bleaches the photopigment. When measured in the non-foveal retina, cone sensitivity recovery occurs first and is very rapid, with rod recovery occurring later, taking up to 45 min for sensitivity to fully recover for a young adult. Studies examining the impact of ageing on dark adaptation were performed in the early 1950s and 1960s [69, 70]. These studies reported impaired light sensitivity during recovery but did not calculate the rate of RMDA recovery (i.e., how rapidly rods recover). These studies also had other methodological limitations. Thresholds were measured infrequently, yielding imprecise parameters in estimating sensitivity recovery parameters; there was no screening or use of an AMD classification system to ensure good macular health, and only the rod-cone break was estimated, not the actual rate of RMDA. Gregory Jackson tackled this topic in his PhD dissertation, overcoming these methodological challenges. Sensitivity was measured frequently during recovery, and the use of an AMD classification system allowed study of older adults in verified normal macular health [71]. An important feature of this study is the use of an objective statistical technique to estimate dark adaptation parameters [72], instead of ‘hand-fitting’ the function by visual inspection.

Results were novel in that with increasing age during adulthood, adults experience substantial delays in the time it takes for rods to adapt to darkness [71]. For example, as compared to younger adults, adults in their 70s and above displayed a rod-cone break that was delayed by about 2 min. Further, the second and third components of rod sensitivity recovery, which estimate the speed of dark adaptation, were also delayed. Finally, the time it takes 70-year-olds to reach their pre-bleach sensitivity was over 10 min longer than for those in their 20s. These findings demonstrate that the ageing process slows the speed of dark adaptation.

## Rod-Mediated Dark Adaptation: AMD

We next addressed the question of whether the emergence of AMD in older adults further exacerbates delays in RMDA. In addition to being aware that rod density is reduced in AMD [21], we also noted a study by Steinmetz and Bird [73] showing that the second component of rod recovery is slow in spite of participants having good visual acuity and steady state scotopic sensitivity. The authors attributed this effect to a localised shortage of vitamin A caused by impaired exchange across Bruch’s membrane or progressive metabolic impairment of the RPE. These findings were intriguing, although we recognised that the sample was small and the AMD and normal control patient determinations were based on clinical impressions, not fundus grading and standardised classifications. We sought to replicate the study in two separate investigations using a fundus classification system. In each study, we found that early or intermediate AMD eyes had slow RMDA at 12° on the superior vertical meridian as compared to older adults in normal macular health [74, 75]. We also noted that while cone-mediated dark adaptation delays were slightly delayed in some eyes, the rod-mediated delays were much more dramatic [75].

Steinmetz and Bird’s hypothesis intrigued us in that a scarcity of vitamin A caused by sub-RPE deposits could lead to a diffusion barrier that disrupts metabolic exchange between the choroid and photoreceptors, thus creating a delay in RMDA. Sorsby’s fundus dystrophy (SFD) [76] is a rare human genetic disease that has structural and functional similarities to AMD, in that extracellular material accumulates between the receptors and choroid, and rod-mediated light sensitivity is impaired. Jacobson and colleagues [77] administered retinol (vitamin A) to several SFD patients, and interestingly, this led to reversals in dark adaptation delays. This raised the question as to whether administration of vitamin A in older and early AMD patients could force more vitamin A to photoreceptors, resulting in a speeding-up of their dark adaptation. We performed a double-masked, double-blind, placebo-controlled experiment administering a short-term (30-day), high-dose of retinol (50,000 international units) to 104 older adults in normal macular health and early AMD [78]. Dark adaptation was measured before and after the course of retinol, and several parameters were computed [72]. We found that the only parameter of dark adaptation that improved was the slope of the second component, which became steeper. There were no changes in the cone time constant, cone sensitivity, the rod-cone break, or rod sensitivity [78]. These findings are consistent with the hypothesis originally expressed by Steinmetz and Bird [73] that a localised shortage of vitamin A caused by sub-RPE deposits causes a diffusion barrier that disrupts metabolic exchange between the choroid and the photoreceptors. Using the Low Luminance Questionnaire (LLQ) previously developed by our group [79], we found that those patients who had greater increases in rod slope reported more improvement on the mobility subscale of the LLQ. That is, patients’ self-reported behaviour reflected the speeding up of RMDA.

## A New Metric for Measuring RMDA

**Fig. 4:**
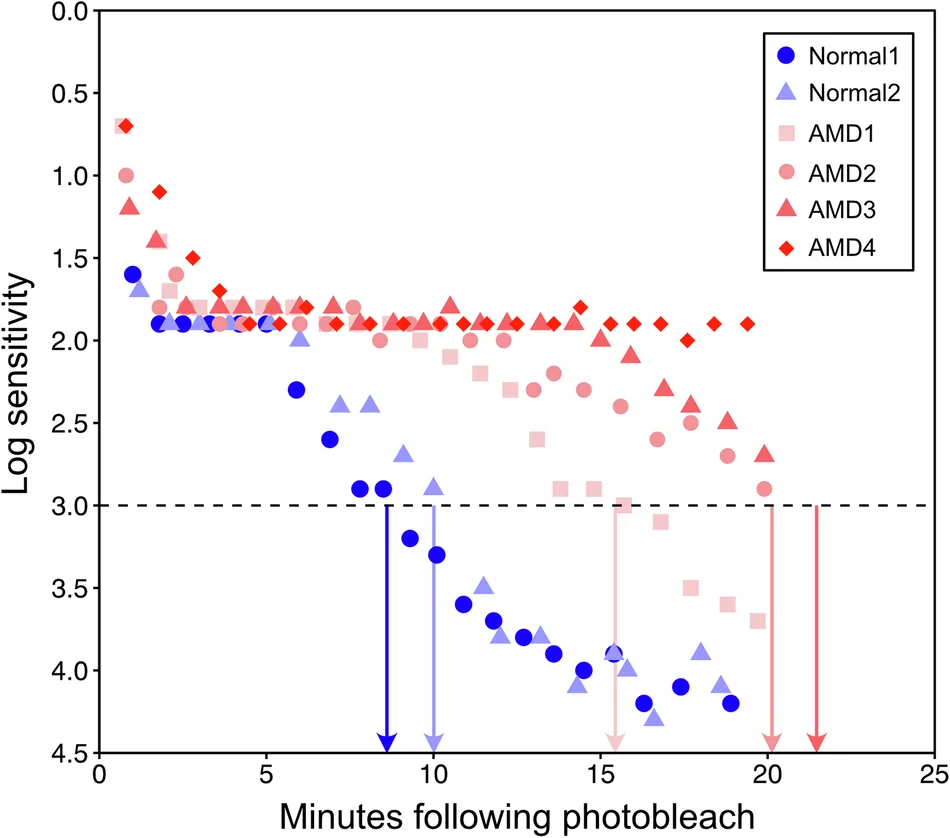
Rod-medicated dark adaptation in normal ageing and age-related macular degeneration. With our colleague Gregory Jackson, we developed a new metric for rod-mediated dark adaptation, called the rod intercept time (RIT). Computing the slope of the second component from dark adaptation data requires model-fitting [72], but using RIT as a surrogate for the slope of the second rod component was much simpler. During dark adaptation testing, RIT is the duration required for sensitivity to recover to a criterion sensitivity value in the latter half of the second component of recovery [80]. This sensitivity criterion is illustrated by the horizontal dotted line at 3 log units of sensitivity. The arrows descending to the *x*-axis illustrate RIT. Higher RIT indicates slower recovery. Representative plots from two older adults in normal macular health and four with early age-related macular degeneration (AMD), depicting the time-course of recovery for a target at 5 degrees following a photo-bleach of 83%. The early component of cone-mediated recovery is followed by rod-mediated sensitivity recovery. Note the dramatically slowed rod-mediated recovery in early AMD eyes compared to those in normal macular health. In some early AMD eyes, they did not recover sensitivity during the 20-min period; some AMD eyes take much longer than 20 min to recover. Used with permission from the Association for Research in Vision and Ophthalmology through the Creative Commons.

With Gregory Jackson, we noticed that there were no computerised devices for measuring RMDA in research or in the clinic. The Goldmann–Weekers Adaptometer, developed in the 1940s, had become the clinical standard, but dark adaptation plots were generated manually. In 2004, we developed and patented a new computerised dark adaptometer called the AdaptDx, a desktop system, which was licensed to a company and ultimately manufactured beginning in 2014. This adaptometer became widely available, especially for conducting research on early and intermediate AMD (for example [80–84]), demonstrating a slowing in RMDA in early and intermediate AMD. An important feature of the AdaptDx is a novel method of measuring the rate of dark adaptation that is available immediately after testing, rather than relying on non-linear regression models to later generate the second component of rod recovery. The new metric was called rod-intercept time (RIT). RIT is defined as the duration required for sensitivity to recover to a criterion sensitivity value in the latter half of the second component of rod recovery [85]. Figure 4 illustrates how eyes with early AMD had prolonged RITs, whereas eyes in normal macular health had briefer RITs. Unfortunately, the company manufacturing the tabletop AdaptDx is no longer operational, so the tabletop device is currently not available. However, the AdaptDx’s lasting contribution is that it established that delayed RMDA is a functional hallmark for incident early AMD and progression to intermediate AMD [86], which will be discussed later. Fortunately, other companies have been developing new computerised dark adaptometers, adopting the AdaptDx’s RIT metric as a key outcome.

## Alabama Study on Early Age-Related Maculopathy (ALSTAR)

ALSTAR was a longitudinal study specifically designed to ask whether RMDA in older adults with normal macular health appears before early AMD is clinically visible in colour fundus photographs. We asked whether slowed RMDA in normal older adults at the baseline visit was associated with the incident development of AMD 3 years later. We noted that at the baseline visit in ALSTAR, 22% of older adults in normal macular health had significantly longer RMDA as compared to the rest of the sample [87]. We wondered if this functional delay could serve as a risk factor, or marker, for incipient AMD, which is funduscopically invisible to the clinician. This hypothesis is attractive because age-related rod loss in the macula [20] is tightly centred around the fovea, where drusen and basal linear deposit preferentially accumulate in ageing and AMD.

One eye was tested at baseline for 325 older adults in normal macular health, 263 eyes having normal dark adaptation, and 62 having delayed RMDA (defined as RIT ≥ 12.3 min). We evaluated participants at baseline and 3 years later, using the Age-Related Eye Disease Studies (AREDS) 9-step AMD classification system [88] to define eyes in normal macular health and those with AMD. Results confirmed our hypothesis [80]. Eyes in normal macular health at baseline with delayed dark adaptation were two times more likely to have incident AMD 3 years later, as compared to those who did not have delayed dark adaptation. This association was adjusted for age and smoking status. The association of other visual functions at baseline with incident AMD at follow-up was also investigated, including photopic, visual acuity, mesopic acuity, low luminance deficit, contrast sensitivity, and photopic light sensitivity. But they lacked significant association except for low luminance visual acuity, which was weaker than that for RMDA [89]. Our study demonstrated that delayed RMDA is the first functional biomarker for incident AMD. Currently, there are no useful functional endpoint measures available for trials evaluating strategies for AMD prevention or interventions to arrest its progression at early stages (even if there were such candidate treatments). Visual acuity is the accepted standard endpoint for preventing or slowing exudative AMD, yet acuity is largely undisturbed during early disease [22, 23]. Thus, one practical importance of our result is that RMDA testing now has demonstrated potential as a functional endpoint for evaluating treatments to prevent early AMD.

## Alabama Study on Early Age-Related Macular Degeneration 2 (ALSTAR2)

**Fig. 5:**
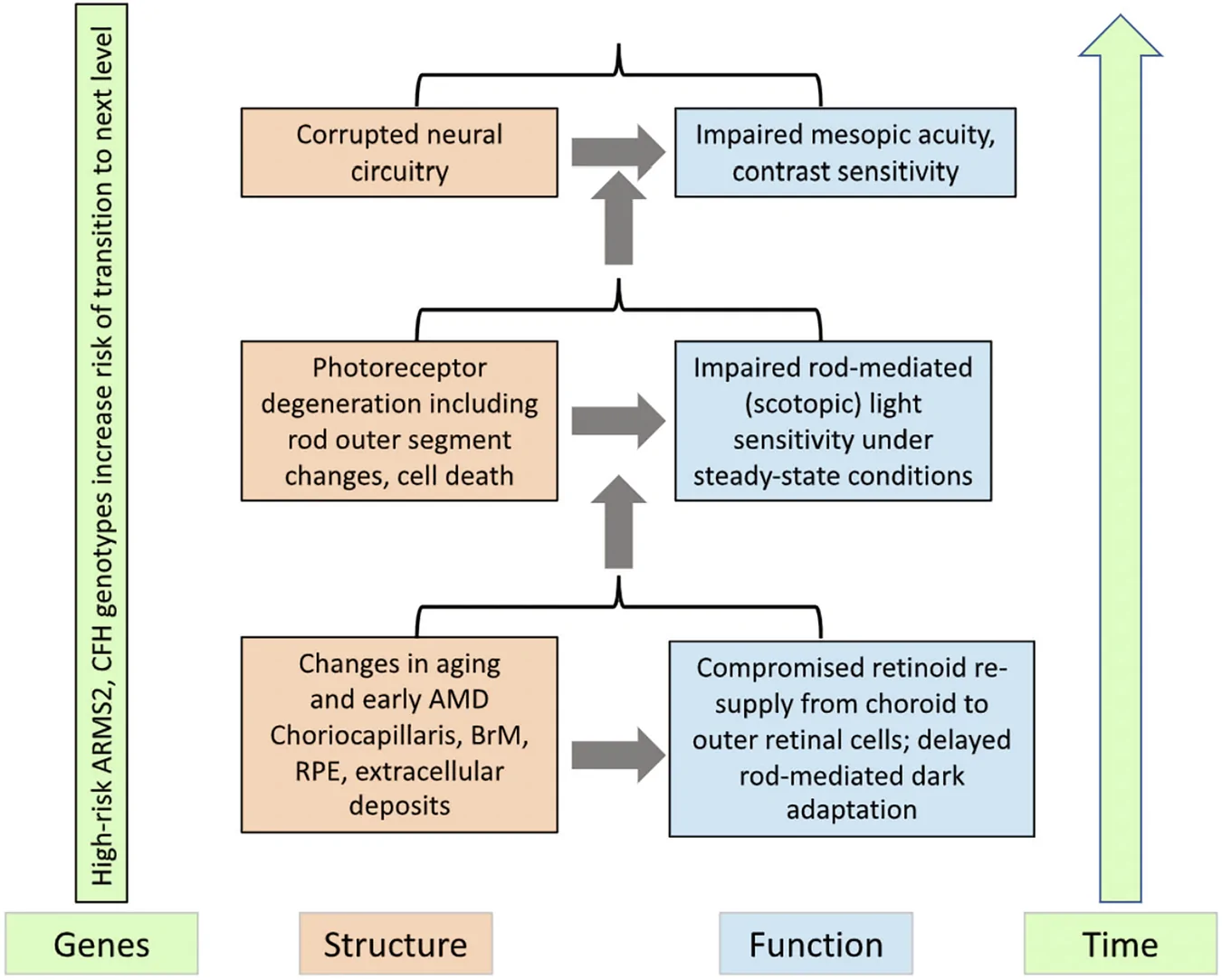
Theoretical framework for how vision is impaired: Ageing to early and intermediate age-related macular degeneration (AMD). Our theoretical framework for vision loss progression is illustrated in terms of how structural changes could be associated with functional changes. At the bottom on the structural side in normal ageing, there are changes in the choriocapillaris, Bruch’s membrane and retinal pigment epithelium, and the emergence of extracellular deposits. This corresponds on the functional side to compromises in retinoid re-supply from the choroid to outer retinal cells, which leads to delayed rod-mediated dark adaptation. When there is progression to early AMD, rod photoreceptor degeneration occurs, with changes to rod outer segments and eventually cell death. Functionally, this results in loss of scotopic sensitivity deficits under steady state conditions (after the eye adapts to darkness). Finally, as intermediate AMD emerges, particularly in the later stages of intermediate AMD (at the top of Figure), neural circuitry is corrupted, leading to deficits in pattern (spatial) vision, including impaired visual acuity and contrast sensitivity. Used with permission of Springer Nature under Creative Commons license; article published here [27]. BrM Bruch’s Membrane, CFH complement factor H, RPE retinal pigment epithelium.

In our next longitudinal study, ALSTAR2 [19], we focused on identifying visual functions in ageing and early and intermediate AMD that can be structurally validated through retinal imaging. Our premise was that the strongest endpoints for use in trials to prevent AMD or to arrest its earliest progression will be functional and structural characteristics that are biologically related to one another. At baseline, we enrolled participants in normal macular health, early AMD, and intermediate AMD, once again using the AREDS 9-step classification system [88]. At baseline and 3-year follow-up visits, we assessed nine visual functions focusing on rod-mediated (including RMDA), cone-mediated, and rod- and cone-mediated vision. Specifically, we measured RMDA at 5°, photopic and mesopic acuity, photopic and mesopic contrast sensitivity, and photopic, mesopic, and scotopic light sensitivity. The retinal imaging protocol was also comprehensive, including colour fundus photography (reflectance), true colour fundus photography (confocal scanning), OCT, OCT angiography, quantitative autofluorescence, 2-wavelength autofluorescence, and fluorescence lifetime imaging ophthalmoscopy. Before beginning the study, we developed a theoretical framework for vision loss progression during normal ageing to early and intermediate AMD based on structure and function (Fig. 5) [19, 22, 27]. Beginning with structure in the eyes with normal macular health, there are ageing-related changes in the choriocapillaris, Bruch’s membrane, RPE, and the beginning formation of extracellular deposits. Functionally, this compromises retinoid resupply from the choroid to outer retinal cells, which generates delays in RMDA. As time progresses, there are rod outer segment changes and cell death leading to photoreceptor degeneration, the basis of early AMD. This compromises rod-mediated (scotopic) sensitivity measured under steady-state conditions. Finally, in intermediate AMD, neural circuitry becomes corrupted, leading to impairments in pattern vision, such as visual acuity and contrast sensitivity.

## ALSTAR2: Cross-Sectional Findings

**Fig. 6:**
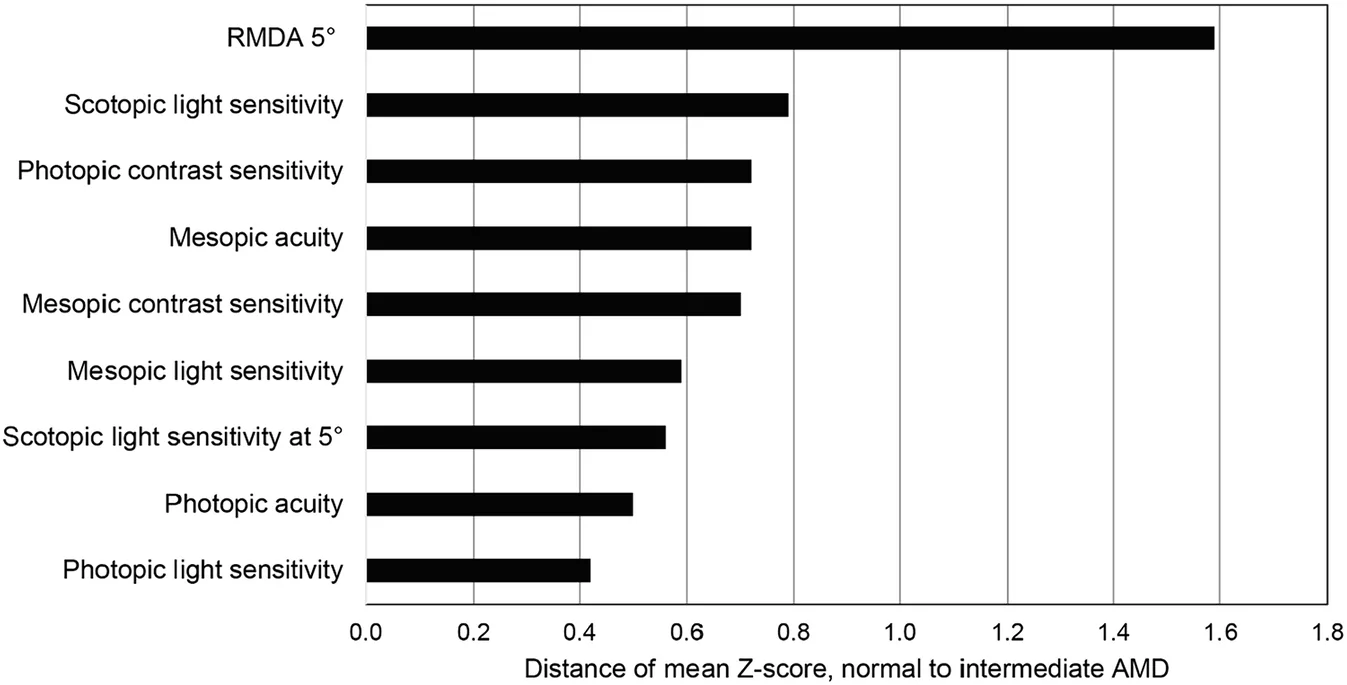
The difference in z-scores for visual function tests in normal ageing and intermediate age-related macular degeneration (AMD). The data for each visual function in baseline Alabama Study on Age-related Macular Degeneration 2 (ALSTAR2) were expressed as z-scores. This figure shows the z-score difference between normal ageing and intermediate age-related macular degeneration (AMD). The largest difference in function by AMD status was for rod-mediated dark adaptation (RMDA) measured at 5°. All z-scores for other visual functions were much smaller, being one-half or less of the range for RMDA 5°. Photopic acuity and light sensitivity were the least likely to separate normal ageing from intermediate AMD. Used with permission from the Association for Research in Vision and Ophthalmology through Creative Commons with article published here [22].

We were first interested in comparing cross-sectional differences at baseline among the three groups of those in normal macular health and having early and intermediate AMD [22]. All visual functions in intermediate AMD were statistically worse than eyes in normal macular health. However, the only visual function that was worse in early AMD, compared to normal macular health, was RMDA, again supporting the notion that it is the first functional biomarker, at least cross-sectionally. The data for each visual function were expressed as z-scores based on the overall distribution of scores across all groups (normal, early and intermediate AMD) and then summarised for each group. Figure 6 shows the z-score difference from normal to intermediate AMD eyes. The largest difference in function in AMD, by far, was for RMDA. The z-score differences for all other visual functions were much smaller, being one-half or less of the range for RMDA. These results also demonstrate that the earliest visual dysfunction to emerge in AMD is delayed RMDA, as analysed cross-sectionally.

## Subretinal Drusenoid Deposit, an AMD Newcomer

**Fig. 7:**
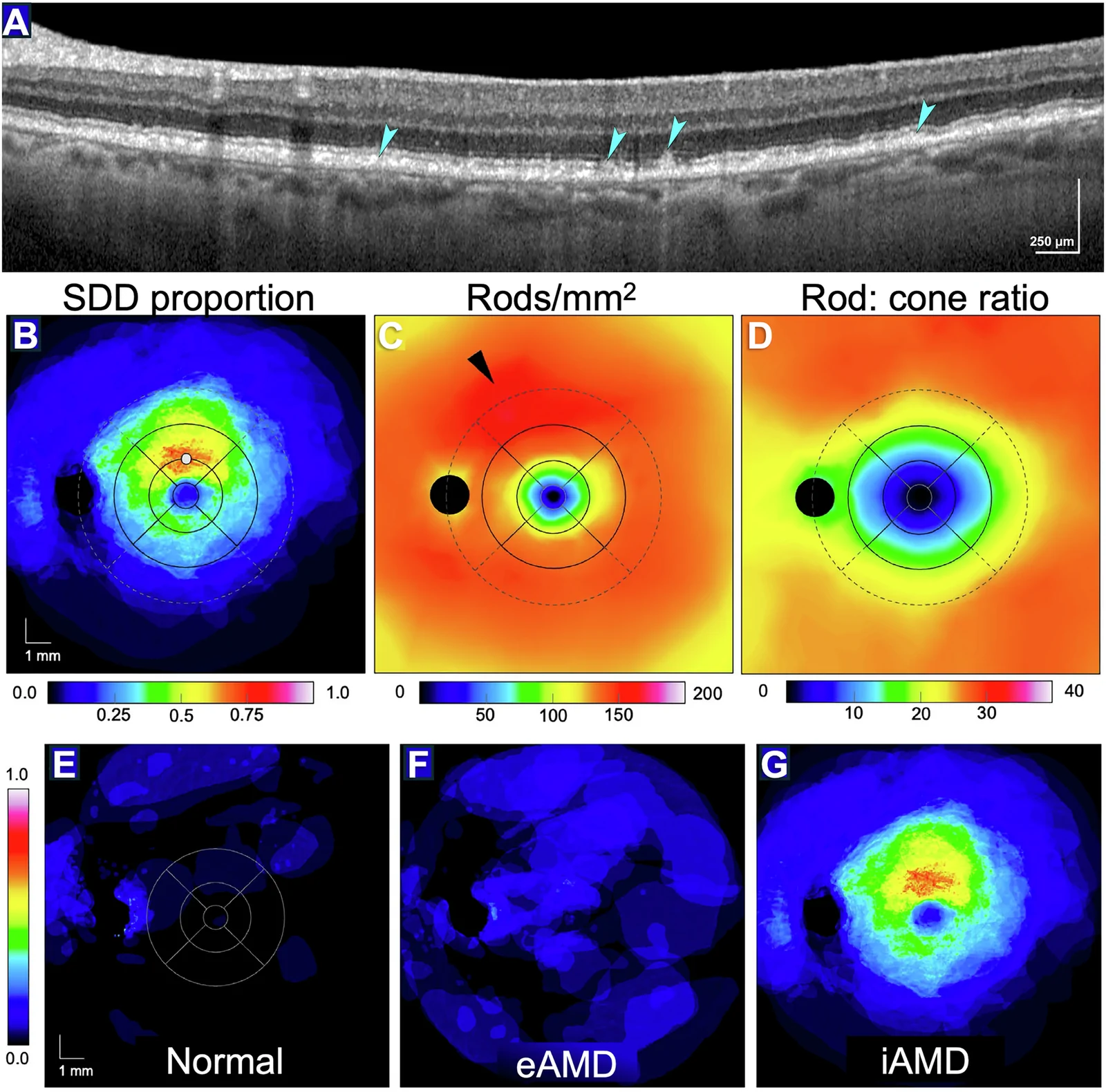
Topography of subretinal drusenoid deposits implicates rods and more. Subretinal drusenoid deposits (SDD) is abundant closer to the fovea than the rod peak and falls off more quickly with eccentricity than the ratio of rods to cones. **A** Spectral domain optical coherence tomography (OCT) shows individual SDD (arrowheads) between the retinal pigment epithelium-Bruch’s Membrane (BrM) band and the ellipsoid zone (EZ). **B**–**D** Comparing the distributions of SDD and rod photoreceptors. **B** Distribution of SDD in older adults (normal and age-related macular degeneration (AMD) together), determined in 55° wide near-infrared reflectance images that are large enough to include the ring of high-rod density [105]. SDD are present in an asymmetrical ring around the fovea, extending more superiorly than inferiorly, and peaking at the test target location for rod-mediated dark adaptation (RMDA) 5° in superior retina. **C** Spatial density of rods in adult human retina. **D** Rod:cone ratio in adult human retina. Distribution of SDD in healthy aged (**E**), early AMD (**F**), and intermediate AMD (**G**) eyes. **E**, **F** SDD manifest first near the optic nerve head (ONH) with a light sprinkling across the central area. **G** The highest proportion of eyes with SDD are iAMD. SDD extend beyond the ETDRS grid and to the nasal side of the ONH. Colour scale: Proportion of eyes in each group showing SDD, determined on a per pixel basis. ETDRS early treatment diabetic retinopathy study, iAMD intermediate age-related macular degeneration, eAMD early age-related macular degeneration. Data for **B**, **C** came from cell counts in flat-mounted donor retinas [28]. Data for **B**–**G** from baseline visit of the Alabama Study on Age-related Macular Degeneration 2 (ALSTAR2), adapted from [105].

The earlier section on laboratory research highlighted the all-cone fovea, the vulnerable rods next to it, and dysregulation of transport between circulation and outer retinal cells that is particularly noticeable under the fovea. A new chapter opened when spectral domain OCT revealed reflectivity between the RPE and the photoreceptors [90] (Fig. 7A) corresponding to material previously seen but not named by Sarks et al. in their microscopy studies [91]. With increasing degrees of thickness and abundance, photoreceptors are increasingly perturbed by the intrusion of this material (subretinal drusenoid deposit (SDD)) [90, 92–95]. Histologic SDD is thick where rods are numerous, and drusen material is thick where cones are numerous [93], a distribution later confirmed in intermediate AMD patients by en face OCT [96]. We proposed that deposits represented dysregulation of lipid transfers serving specifically cone and rod outer segment membrane maintenance. However, the relation to ageing was not as clear for SDD as it was for high-risk drusen. There were no apparent tissue-level precursors analogous to the lipoproteins in Bruch’s membrane. However, persons with SDD tended to be older than persons without. In 2015, Catherine Cukras and the clinical research team at the US National Eye Institute showed that eyes with SDD (called reticular pseudodrusen in en face viewing) had extremely delayed RMDA 5° [81].

**Fig. 8:**
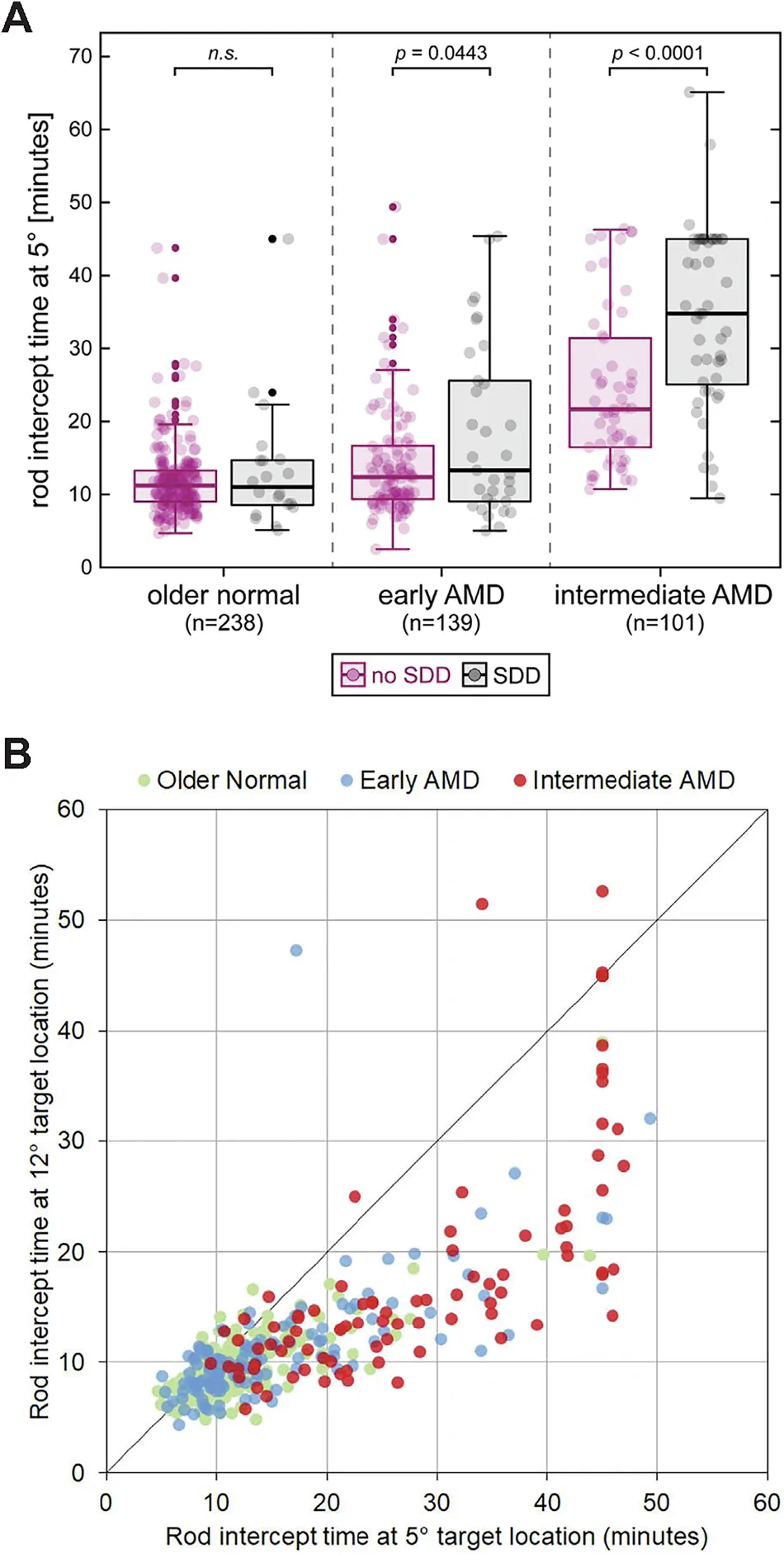
The impact of subretinal drusenoid deposits (SDD) and eccentricity of test target on rod-mediated dark adaptation (RMDA). **A** Rod-intercept time (RIT) is stratified by disease severity and subretinal drusenoid deposits (SDD) absence versus SDD presence in older normals, early age-related macular degeneration (AMD), and intermediate AMD eyes. Circles are data points from individual eyes. Box-and-whisker plots represent median and interquartile ranges. Early and intermediate AMD eyes with SDD present had longer RITs than eyes with SDD absent. Yet in these eyes, SDD absent eyes also exhibited delayed RMDA. SDD grading was based on near-infrared reflectance and also en face and B-scan multimodal imaging as discussed in [22]. AMD severity groups are assigned based on colour fundus photography grading (ARED 9-step). Used with permission from the Association for Research in Vision and Ophthalmology with article published here [22]. **B** Scatterplot showing RIT at 5° and 12° for each participant stratified by the Age-Related Eye Disease Studies (AREDS) 9-step system (normal ageing, early AMD, intermediate AMD). The identity line represents a hypothetical scenario in which RMDA at 5° and 12° are equal. The majority had longer RIT at 5° than at 12°. RIT for RMDA at 5° in intermediate AMD eyes is ≥9.5 min whereas RIT in many older normal and early AMD eyes is <9.5 min. Used with permission from the Association for Research in Vision and Ophthalmology through Creative Commons with article published here [100].

The presence of SDD increases the risk for end-stage AMD [97], and thus, it is an important characteristic to probe for understanding prognosis. Because of this prior report [81], which was replicated by other groups [82, 98, 99], we wished to check how SDD impacted rod vision in the ALSTAR2 cohort. Within the context of ALSTAR2 and our very large sample of *N* = 481 eyes, including many normal aged eyes, the impact of SDD on RIT was examined in all three groups (i.e., normal, early and intermediate AMD) [22], compared to eyes with no SDD. In the normal group, SDD had no impact on RIT. This result is not surprising, since SDDs are very small, if present in the eyes in normal macular health (Fig. 8A) [22]. However, in early and intermediate AMD, RIT was significantly longer as compared to eyes without SDD, with the greatest impact in intermediate eyes [22].

Because RMDA is assessed outside the fovea, we evaluated the impact of target location on normal ageing and early and intermediate AMD [100]. We hypothesised that RMDA would be more delayed when tested at a retinal location near mechanisms leading to or resulting from high-risk extracellular deposits, i.e., near the fovea where these deposits occur, versus further away from the fovea. However, further away from the fovea, SDDs are much more common. In testing older adults with and without early and intermediate AMD, RMDA was tested at 5°, where rod loss is prevalent and at 12°, where rod density is the highest [20, 21]. Our hypothesis was confirmed in that RIT is significantly longer (i.e., RMDA is slower) at 5° than at 12° for each AMD severity group [100] (Fig. 8B). The impact of the longer RIT is greatest with greater disease severity, that is, in intermediate AMD. Other research groups have also found slower RMDA closer to the fovea in AMD as compared to more eccentric locations [101–104]. Thus, proximity to the fovea has a strong and repeatable effect in slowing RMDA.

Previous functional studies of eyes with SDD included mostly intermediate AMD, so to determine how the SDD distribution evolves from ageing to AMD, we turned to clinical imaging (Fig. 7). In the ALSTAR2 baseline, we quantified SDD extent images wide enough to capture the rod ring of high rod density (55° wide near-infrared reflectance), nearly 7X larger than the ETDRS grid [105]. The extent of SDD is moderately and specifically correlated with RMDA 5° [105]. We, like others, found that SDDs are abundant in the superior outer ETDRS subfield, but we also found that the SDD peak is closer to the fovea than the peak of rod density (Fig. 7B–D). In fact, the RMDA 5° target location, chosen years ago because of age-related rod loss, precisely probes the location of future SDD. In older and early AMD eyes [105], SDD appears at the optic nerve head, where the choroid is thin, with a light sprinkling of SDD throughout the central area (Fig. 7E–G). In this cohort, eyes with intermediate AMD account for most of the SDD. So SDD does appear where rods are numerous, but the distribution also reveals vascular origins (in ageing) and proximity to the fovea (with later disease) as important factors.

**Fig. 9:**
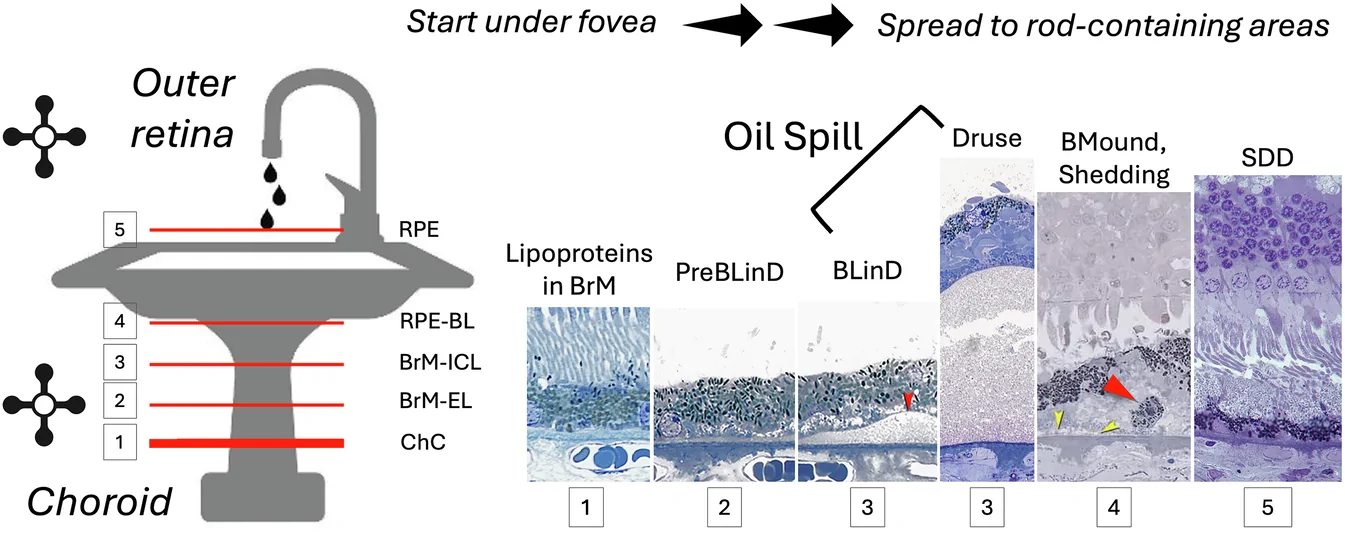
Sequential barrier of age-related macular degeneration (AMD) deposit formation, heralded by delayed rod-mediated dark adaptation (RMDA) 5°. A unified theory of AMD deposit formation posits sequential barriers to outflow of metabolites from the outer retina that move inward over time, starting with Bruch’s membrane (BrM) and choriocapillaris (ChC) under the fovea. Delayed RMDA 5°, which starts in ageing, potentially indicates dysfunctional transport before this pathology is visible in the fundus. We propose that two dials (at the left) control the formation of extracellular deposits. The top one represents physiologic release of material from outer retinal cells. The bottom one represents physiologic flow-control to circulation. The faucet and drain respectively represent generation of metabolites and outflow to the choroid. We propose (based on deposit composition and experimental studies) that this outflow evolved to constitutively dispose of materials generated by or are left over from dietary supply to photoreceptors. Deposits form where metabolic needs are high (e.g., where cells are numerous at the fovea or rod ring) and/ or transport to circulation is impaired. Five planes of dysregulated outflow (numbered 1–5) start at the ChC (endothelium, flow, or both) and move inward over time. High-resolution histology illustrates five layers of deposited materials corresponding to barriers 1–5: (1) lipoproteins in ageing BrM (blocked by BrM and ChC); (2) pre-basal linear deposit (BLinD) (also known as the Lipid Wall, blocked by Bruch’s membrane – elastic layer (BrM-EL)) (3) Oil Spill components soft drusen and BLinD (blocked BrM-EL); (4) soft drusen material trapped within the basal laminar deposit (BLamD), i.e., basal mound (BMounds - blocked by the RPE-basal lamina (RPE-BL)); (5) SDD, blocked by RPE cell bodies. We propose that transport dysregulation spreads outward to rod-containing areas over time during ageing. Only the Oil Spill and its precursors, starting under the fovea in late adolescence with lipoprotein deposits, is specific to AMD. Both BLamD and SDD occur in many disorders; SDD occurs in slightly older persons than do drusen. BrM-ICL Bruch’s membrane inner collagenous layer, RPE retinal pigment epithelium, SDD subretinal drusenoid deposits,  Reprinted from Proctor Medal Lecture [27], which has all the references.

Our current hypothesis is that RMDA captures dysregulation of transport starting in late adolescence and extending throughout adulthood, and that SDD is a late manifestation of that dysregulation. A unified model of AMD deposit formation is now possible (Fig. 9). Let us propose two dials to control deposit formation. One is the constitutive release of material from outer retinal cells. The other is the flow control of output to circulation. Deposits form where metabolic needs are high (e.g., fovea and rod ring where cells are densely packed) and/ or transport to circulation is impaired. We propose that outflow evolved to dispose of materials generated primarily by or leftover from dietary supply to photoreceptors. Outer segments form new disk membranes every 20 min [106]. On the left of Fig. 9 are five planes of dysregulated outflow, starting at the choriocapillaris endothelium and moving inward over time. On the right are five layers of materials, starting with lipoproteins in ageing Bruch’s membrane, soft drusen and precursors (Oil Spill), and soft drusen material trapped within basal laminar deposit (basal mounds). Finally, when the RPE itself is the barrier, SDD appear. We propose that this process starts at the fovea and spreads outward to rod-containing areas over time. Thus, delayed RMDA 5°, which starts in ageing, potentially indicates dysfunction before this pathology is visible. The model incorporates both the specificity of high-risk drusen for AMD and the relative non-specificity of other deposits (basal laminar deposit and SDD), which occur in other diseases.

## ALSTAR2: Longitudinal Findings

The longitudinal findings in ALSTAR2 between baseline and 3-year follow-up provided us with the opportunity to examine the incidence of AMD in eyes that were normal at baseline in terms of six visual functions (scotopic light sensitivity, mesopic contrast sensitivity, mesopic light sensitivity, low luminance visual acuity, photopic contrast sensitivity, and photopic visual acuity. Additionally, to determine which visual functions were associated with incident AMD 3 years later. The study also stimulated the question of whether RMDA and the six visual functions in early AMD were associated with the progression of early AMD 3 years later.

**Fig. 10:**
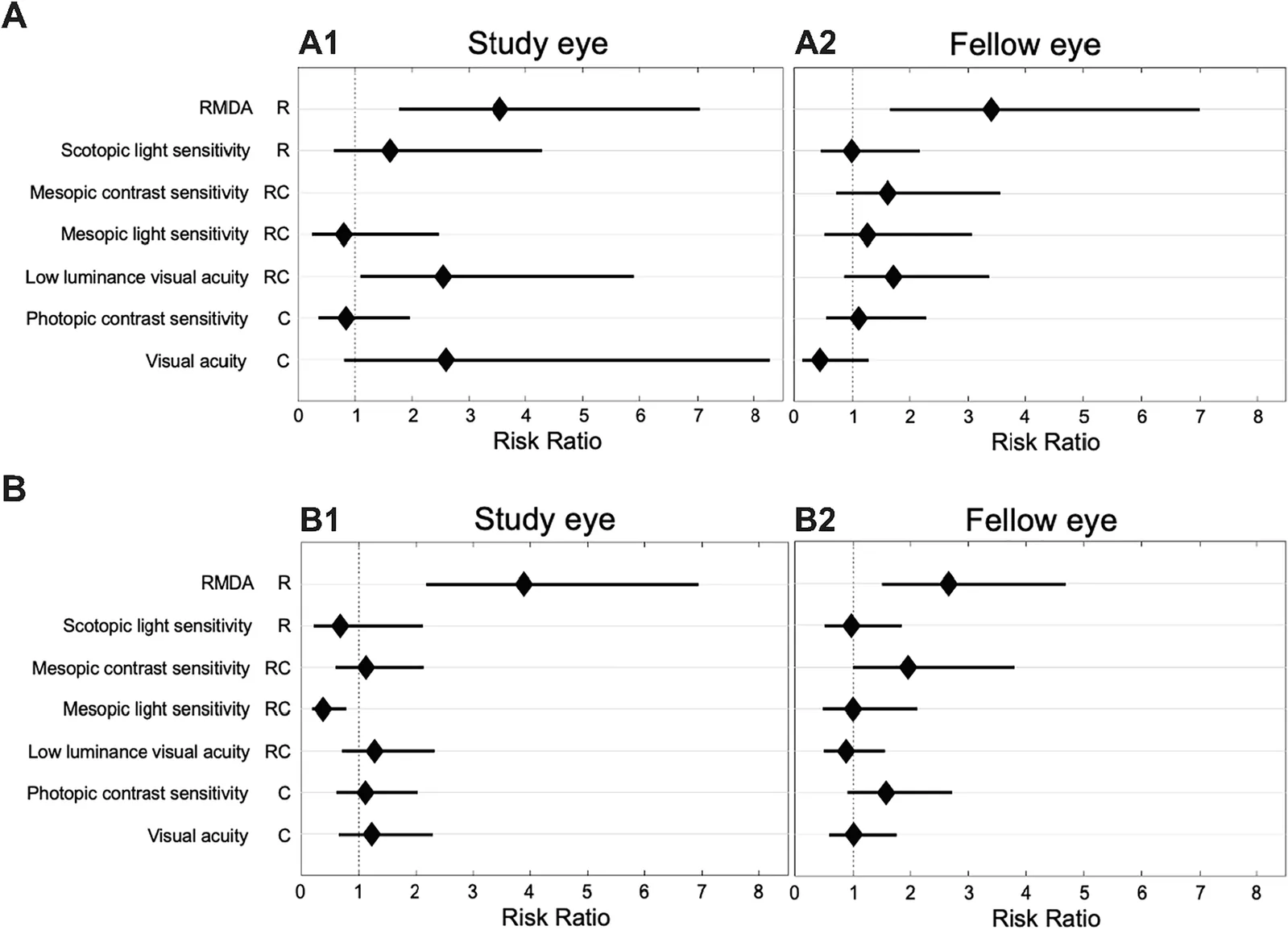
Risk ratios and 95% confidence intervals for incidence and progression of age-related macular degeneration (AMD) for all visual functions in the Alabama Study on Age-related Macular Degeneration 2 (ALSTAR2). **A1** Plot showing the incidence of age-related macular degeneration (AMD) in eyes at the 3-year follow-up that are healthy at baseline, stratified by visual function at baseline. **A2** Similar plot showing the incidence at follow-up but for the fellow eye. Note that rod-mediated dark adaptation (RMDA) has the strongest and almost the sole relationship for understanding risk for AMD incidence. **B1**, **B2**. This is identical to A1 and A2, but this is for eyes that were early AMD at baseline. Note that only RMDA is associated with the progression of AMD in eyes that had early AMD at baseline. This is true for the study eye and the fellow eye. All these figures published with permission from Elsevier with the article published here [86]. R, RC and C indicate rod-, rod and cone- and cone-mediated functions, respectively.

The risk ratios (RR) and associated 95% confidence intervals for incident AMD [86] are shown in Fig. 10A. The Youden index [107] was used to generate visual function cut-points for understanding risk. The main finding is that the only baseline visual function strongly associated with incident AMD in the tested eye 3 years later is RMDA, with delayed dark adaptation being 3.54 times more likely to have AMD at follow-up [86]. It was also interesting that RMDA was associated with incident AMD in the fellow eye with a very similar RR (3.40). Low luminance acuity had a weaker association with an RR of 2.54; however, it was not associated with progression of AMD in the fellow eye. No other visual functions were associated with progression.

The results were very similar for eyes with early AMD and their association with AMD progression after 3 years (Fig. 10B) [86]. Only RMDA at baseline was associated with the progression of AMD in both the study eye and the fellow eye (RR 3.89 and 2.65, respectively), with no other visual functions associated with progression. In summary, these findings clearly indicate that to understand incident AMD and progression of early AMD, only RMDA provides information about risk. Other visual functions, even though they can be impaired at baseline [22], do not help us understand AMD incidence and progression in early disease.

In our longitudinal ALSTAR2 data set on visual function [108], we were interested in the natural history of changes in visual functions over 3 years in ALSTAR2 for each of our baseline groups: normal macular health, early AMD and intermediate AMD. We wondered whether any observed changes were statistically significant. A second purpose was to see whether any observed changes were minimal clinically important differences (MCID), establishing a threshold for change that is actually meaningful to patients [109, 110]. To address statistically significant changes in visual functions over 3 years, we noted that results depended on the diagnostic group [108]. The visual functions measured longitudinally were best-corrected visual acuity and low luminance visual acuity, photopic and mesopic contrast sensitivity, mesopic and scotopic contrast sensitivity, and RMDA at both 5° and 12 °. For eyes in the normal macular health group, all visual functions worsened over time except for mesopic contrast sensitivity. In early AMD, all visual functions worsened over 3 years except photopic and mesopic contrast sensitivity and scotopic light sensitivity. In intermediate AMD, all visual functions worsened over 3 years.

**Fig. 11:**
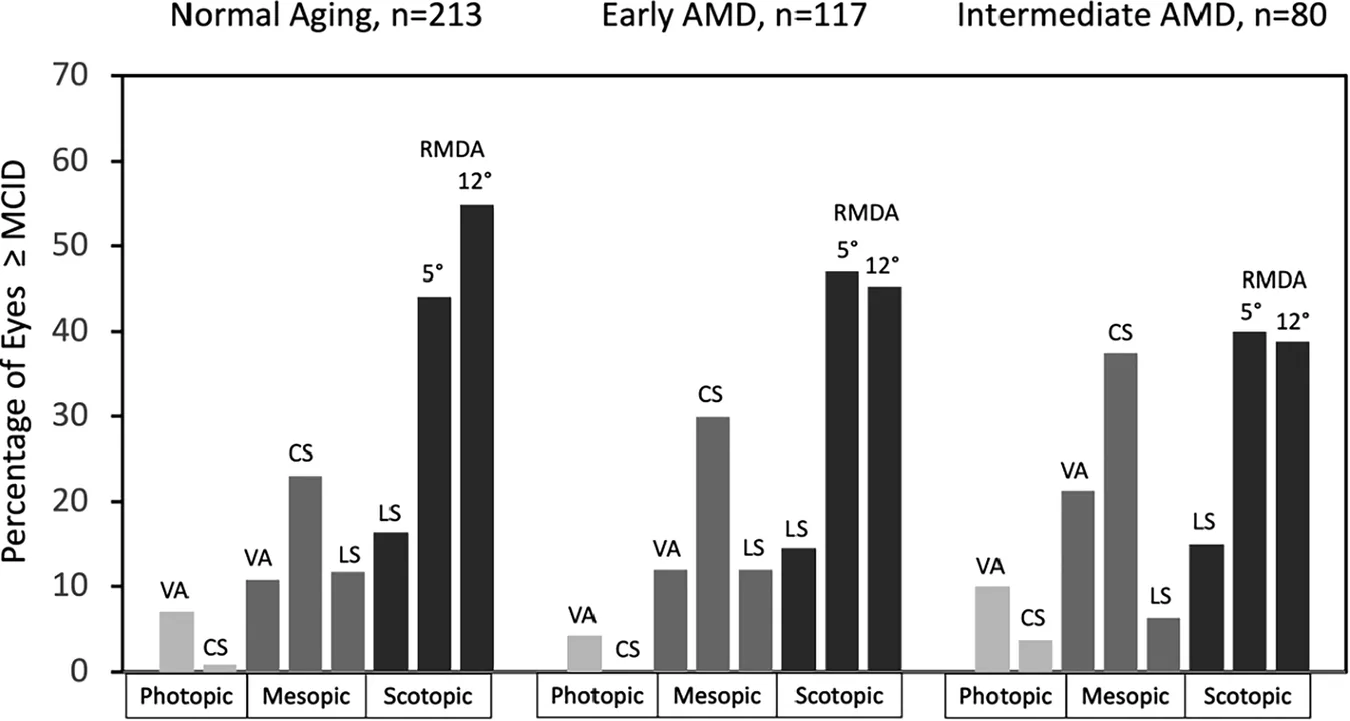
Percentage of eyes that met or went beyond the minimal clinically important difference (MCID) over 3 years, for each visual function. Visual functions included VA (visual acuity), CS (contrast sensitivity), LS (light sensitivity), and rod-mediated dark adaptation (RMDA) measured at 5° and 12°. The largest percentage of eyes meeting or going beyond MCID was for RMDA at 5° and 12°, which ranged from 40% to 54.9%. The lowest percentage of eye meeting or going beyond MCID was for best-corrected visual acuity or photopic contrast sensitivity. Used with permission from the Association for Research in Vision and Ophthalmology through Creative Commons with article published here [108].

Statistical significance is an important benchmark in visual function for understanding change and the magnitude of change over time [111, 112]. By understanding the natural history, clinical trials can identify key timepoints for intervention, optimise treatment timing, and maximise the potential for a positive treatment outcome. Yet MCID is also critically important from the patient’s perspective. An established MCID for visual acuity is 15 letters on the ETDRS chart, which has been accepted by the US FDA [17]. An MCID for photopic contrast sensitivity has been proposed as 0.3 log sensitivity, i.e., a doubling of contrast threshold [113]. The remaining visual functions in ALSTAR2 do not have accepted MCIDs. We used a common distributional method based on the statistical properties of measures [114] used for patient-reported outcome approaches to estimate MCIDs for the other visual functions. A rule of thumb based on prior approaches defines MCID as 0.5 times the standard deviation, which we used for other visual functions [114]. Figure 11 plots the percentage of eyes for each visual function in each group that met or went beyond the MCID. The largest percentage of eyes meeting or going beyond MCID was for RMDA measured at 5° and 12°, which ranged from 40% to 54.9% depending on the group. The lowest percentage of eyes meeting or going beyond MCID was for best-corrected visual acuity and photopic contrast sensitivity, ranging from 0% to 10%. The remaining visual functions fell in between these two extremes with respect to the percentage of eyes that met the MCID. Thus, RMDA demonstrates the strongest relevance in normal ageing, early and intermediate AMD for understanding MCID from the patient’s perspective. One recommendation to scientists proposing a visual function for use in a trial is that it is important to ensure that the proposed visual function has an established MCID, against which a change observed in the trial can be compared.

## Repeatability of RMDA

For RMDA to be an acceptable outcome in trials on early AMD, the repeatability of dark adaptation testing must be established. We designed a single-site longitudinal cohort study examining RMDA at baseline and then again at a second visit 1–2 weeks later [115]. We focused on the same older patient groups as used in ALSTAR2, normal macular health and early and intermediate AMD. We used two different AMD classification systems, the AREDS 9-step system [88] as implemented in the ALSTAR/2 studies, and also the Beckman classification system [116], since the latter system is commonly used in the literature. RMDA was tested at 5° in the vertical meridian, with the identical procedure used in ALSTAR2. The intraclass correlation coefficient (ICC) [117] and 95% confidence intervals were computed for each group and across all groups. For the AREDS 9-step system, for normal ageing, early and intermediate AMD, we found ICCs at excellent levels of 0.92, 0.82 and 0.99, respectively, with an overall ICC for the entire sample of 0.99 [115]. Our results for the Beckman classification overall and per group were highly similar. Our ICC findings are very similar to but slightly higher than reported by Flamendorf et al. [81] and Hess et al. [118], who used a similar methodology. Overall, RMDA, as measured at 5°, is a repeatable test and appropriate for use in trials and observational studies with these populations. An important point to emphasise is that coordinators who collect this data must be very well-trained, demonstrating over repeated testing, that they are effective in administering the test. Also, they must facilitate patients’ understanding that testing will not be brief, so as to prepare the patient psychologically for the length of the test.

## Impact of Cataract on RMDA

Cataract is common in older adults, and while some older adults are pseudophakic, many still have cataractous lenses into the later decades of life. In a longitudinal trial, an eye may be cataractous at one visit and pseudophakic at the next visit. We designed a study to examine how an eye’s RIT changes after cataract surgery so it can be compared to before cataract surgery [119]. We used the three patient groups as studied in ALSTAR2 and the repeatability study above, those in normal macular health and early and intermediate AMD. Before surgery, cataract opacity (in terms of nuclear sclerosis, nuclear colour, and cortical) was assessed by a trained grader on a slit lamp using the Lens Opacity Classification System III (LOCS III) [120, 121] after the eye was dilated. LOC III opacity judgments were based on integer scores. Higher grades in the LOCS III score correspond to greater opacity. Nuclear sclerosis grades and nuclear colour grades can vary from 0 to 6, and cortical grades vary from 1 to 5. RMDA was measured as in the ALSTAR2 study. Within patients’ eyes, we compared RIT before and after cataract surgery. On average, RIT was slower (meaning higher RIT) after post-surgery. We separated our group into eyes that had LOCS scores <4 for nuclear sclerosis, nuclear colour, and/or cortical and those that had LOC III scores of ≥4. Eyes with moderate cataract (<4) averaged an increase of RIT of 0.7 min; those with more significant cataract (≥4) averaged an increase in RIT of 3.1 min [119]. Since RIT increases post-cataract surgery were small for eyes with LOC III scores <4 (less than a minute), which is within the test-retest repeatability of RIT, this is not practically meaningful. On the other hand, in those with LOCS III score ≥4, there was a sizeable increase in RIT, averaging 3.1 min. Our recommendation is that eyes with cataract at a moderate level or less (<4 on the LOCS III system) should be eligible for RMDA testing in longitudinal studies. On the other hand, eyes with opacities greater than moderate (i.e., LOCS III ≥ 4) should not be enrolled in longitudinal studies on RMDA, particularly because they may undergo cataract surgery during the study period. We suggest that a parsimonious explanation for our results may be that age-related cataract acts as a filter to reduce the magnitude of photobleaching. Following cataract surgery, the bleach will very likely be magnified, thus generating a longer RIT.

## Future Research

Our future work entails several areas. First, we and regulatory agencies such as the FDA and EMA are interested in determining how slowing of RMDA impacts a patient’s everyday life. RMDA is measured under scotopic conditions, but humans do not engage in many behaviours at scotopic light levels. However, mesopic conditions do rely on rods. Currently, we are examining the association between RIT and reading and mobility under mesopic conditions. Reading and mobility are two of the key behaviours that are often cited as everyday problems in patients with early and late AMD [79, 122].

Second, a frequent criticism of RMDA tests is that they are too long for a clinical trial. We are investigating shortening the test by reducing the photobleach magnitude since the slope of the second component (the predecessor of RIT) is invariant across bleach levels. A weaker bleach could shorten the test. We are soon to embark on a Rasch analysis of the LLQ [79] to determine how practically useful it could be for examining patient-reported outcomes in clinical trials on AMD.

Third, we are continuing to explore imaging biomarkers of degraded rod-mediated vision that might be useful in clinical practice, especially as automated detection of OCT features is becoming commercially available. Based on ALSTAR2 baseline data, the OCT reflective band called the interdigitation zone (IZ), where the RPE apical processes transfer retinoids to the photoreceptor outer segments, is a strong possibility for a very early biomarker [123]. Another biomarker is a metabolic imaging technology for RPE health, fluorescence lifetime imaging ophthalmoscopy [124]. Both modalities showed a strong correlation with delayed RMDA in ALSTAR2 baseline data. Finally, OCT angiography highlighted a stronger correlation of delayed RMDA 5° with choriocapillaris flow deficits under the fovea than with deficits outside the fovea, consistent with, but not proving the overall model of pathophysiology [125, 126].

Finally, recognising that delayed RMDA next to the fovea is the defining visual dysfunction of the ageing-AMD transition can lead to therapeutic breakthroughs. It is reported that two-thirds of recent drug approvals in the US involved mechanisms of action with supportive genetics data [127]. The path from the 2005 genome-wide association studies to approved inhibitors of complement proteins for geographic atrophy is well known. We can anticipate similar success for AMD stages earlier than atrophy and neovascularisation by the discovery and validation of genes underlying delayed RMDA. To date, risk alleles in age-related maculopathy susceptibility locus 2 (*ARMS2)* and complement factor H (*CFH),* the two largest genetic factors for AMD onset, were associated with delayed RMDA and no other tested visual functions, in ALSTAR [128, 129]. Further, in a population-based study, a polygenic risk score was significantly associated with RIT only [130]. Many proteins of genomic significance, such as CFH, localise to the Bruch’s membrane component of the retinoid supply route [129]. Genotyping cohorts with well-defined visual phenotypes like ALSTAR/2 will add much value in the effort to intervene early in AMD or, ideally, prevent it.
